# Evaluation of the potential incidence of COVID-19 and effectiveness of containment measures in Spain: a data-driven approach

**DOI:** 10.1186/s12916-020-01619-5

**Published:** 2020-05-27

**Authors:** Alberto Aleta, Yamir Moreno

**Affiliations:** 1 ISI Foundation, Via Chisola 5, Torino, 10126 Italy; 2 Institute for Biocomputation and Physics of Complex Systems (BIFI), University of Zaragoza, Zaragoza, 50018 Spain; 3 Department of Theoretical Physics, University of Zaragoza, Zaragoza, 50018 Spain

**Keywords:** COVID-19, Metapopulation dynamics, Disease spreading

## Abstract

**Background:**

We are currently experiencing an unprecedented challenge, managing and containing an outbreak of a new coronavirus disease known as COVID-19. While China—where the outbreak started—seems to have been able to contain the growth of the epidemic, different outbreaks are nowadays present in multiple countries. Nonetheless, authorities have taken action and implemented containment measures, even if not everything is known.

**Methods:**

To facilitate this task, we have studied the effect of different containment strategies that can be put into effect. Our work referred initially to the situation in Spain as of February 28, 2020, where a few dozens of cases had been detected, but has been updated to match the current situation as of 13 April. We implemented an SEIR metapopulation model that allows tracing explicitly the spatial spread of the disease through data-driven stochastic simulations.

**Results:**

Our results are in line with the most recent recommendations from the World Health Organization, namely, that the best strategy is the early detection and isolation of individuals with symptoms, followed by interventions and public recommendations aimed at reducing the transmissibility of the disease, which, although might not be sufficient for disease eradication, would produce as a second order effect a delay of several days in the raise of the number of infected cases.

**Conclusions:**

Many quantitative aspects of the natural history of the disease are still unknown, such as the amount of possible asymptomatic spreading or the role of age in both the susceptibility and mortality of the disease. However, preparedness plans and mitigation interventions should be ready for quick and efficacious deployment globally. The scenarios evaluated here through data-driven simulations indicate that measures aimed at reducing individuals’ flow are much less effective than others intended for early case identification and isolation. Therefore, resources should be directed towards detecting as many and as fast as possible the new cases and isolate them.

## Background

The first report by the Chinese authorities of the COVID-19 outbreak appeared in December 31, 2019. Ever since then, the World Health Organization (WHO) and national public health authorities have been tracing and reporting on the evolution of the outbreak. As initially feared, and despite containment measures adopted in China, with a big city like Wuhan being quarantined for weeks, the disease spread beyond mainland China. As of February 29, 2020, there were 85,403 cases worldwide, of which 79,394 corresponded to China [1]. As of April 13, 2020, there are 1,773,084 cases worldwide, of which 166,019 are in Spain [2]. Three months into the outbreak, much is still unknown about the natural history of the disease and the pathogen. Important from the modeling perspective, for instance, it has been claimed that a large number of cases might have gone undetected by routinely screening passengers, due to the special characteristics of this disease [3]. Admittedly, several studies predict that only between 10 and 20% of the cases have been detected and reported [4–7].

As with any other novel disease, governments, public health services, and the scientific community have been working towards stopping the spreading of COVID-19 as soon as possible and with the lowest possible impact on the population [5, 8–10]. From a scientific point of view, there are two courses of action that can be followed. On the one hand, new vaccines and pharmaceutical interventions need to be developed. This usually requires months of work. Therefore, on the other hand, it is important to study the large-scale spatial spreading of the disease through mathematical and computational modeling, which allows evaluating “in silico” what-if scenarios and potential containment measures to stop or delay the disease. This modeling effort is key, as it can contribute to maximize the effectiveness of any protection measures and gain time to develop new drugs or a vaccine to protect the population. Here, we follow the modeling path and analyze, through a data-driven stochastic SEIR metapopulation model, the temporal and spatial transmission of the COVID-19 disease in Spain as well as the expected impact of possible and customary containment measures.

Our model allows to implement and quantify the impact of several conventional strategies in Spain. These policies are mostly aimed at reducing the mobility of individuals, but we also include other plausible settings like a reduction in the time for case detection and isolation. Our findings agree with previous results in the literature that have reported that a reduction as large as 90% in traffic flow has a limited effect on the spreading of the disease. Important enough and at variance with such studies, the data-driven nature of this study and the available dataset allowed us to disentangle the impact of each transportation mode in several scenarios of mobility reduction in Spain. We found that while shutting down completely any transportation means does not lead to a significant reduction in the incidence of the disease, in some contexts, the arrival of the peak of the disease is delayed by several days, which could eventually be advantageous. Altogether, we provide evidences supporting the adoption of a mixed strategy that combines some mobility restrictions with, mainly, the early identification of infectious individuals and their isolation. These conclusions agree with the recommendations by the WHO [11]. We also highlight that although this study has been made with data from Spain, our findings can also be valid for any other country given the ubiquity of mobility patterns worldwide.

Since this article was first submitted in early March, we have learned more about the characteristics of the COVID-19, yet much is still unknown. Despite this, the overall results of this article still hold, since it was deliberately focused on qualitative behaviors rather than precise quantitative predictions. In fact, as of 13 April, it is still unknown the actual reach of the outbreak in Spain. Some estimates indicate that the actual fraction of infected individuals is 20 to 200 times larger than the number of detected cases [12]. As such, any study based on fitting the exact values reported by the authorities would be inherently flawed, even more if we take into account that the actual delay between symptoms’ onset to official registration of the case is 14 days (added to the long period between infection and symptom onset, this implies that current figures are due to infections that took place more than 20 days ago). There are several other issues with the current data provided by the authorities, which we explain in more detail in the supplementary material (Additional file 1: section 4). As a consequence, it is currently not possible to use these figures to fit simple models and produce precise quantitative predictions. In order to do so, not only we need to collect much more information but also a great deal of forensic data analysis will have to be performed to curate the data—something beyond the scope of this early assessment. Nevertheless, there are some characteristics of this outbreak that can be already analyzed, even with this simplified modeling framework, and we have included some comments related to such observations.

## Methods

Stochastic SEIR metapopulation models are routinely used to study the temporal and spatial transmission of diseases like the COVID-19 [13]. Here, we make use of such class of models and implement a data-driven version that allows obtaining realistic estimates for the spatial incidence of the disease as well as its temporal dynamics. More specifically, in terms of time, we feed the model with the available data as of February 28, 2020. Spatially, we consider that each province (there are 52 in Spain, see Additional file 1: section 2) [14, 15] is represented by a subpopulation. Furthermore, metapopulation models are composed by two types of dynamics: the disease dynamics governed by the chosen compartmental model, SEIR in our case, and the mobility of the individuals across the subpopulations that make up the whole metapopulation system. The latter ingredient, the mobility, connects the subpopulations and allows the disease to spread from one subpopulation to another. In what follows, we describe these two components of our model.

### Mobility dynamics

**Fig. 1:**
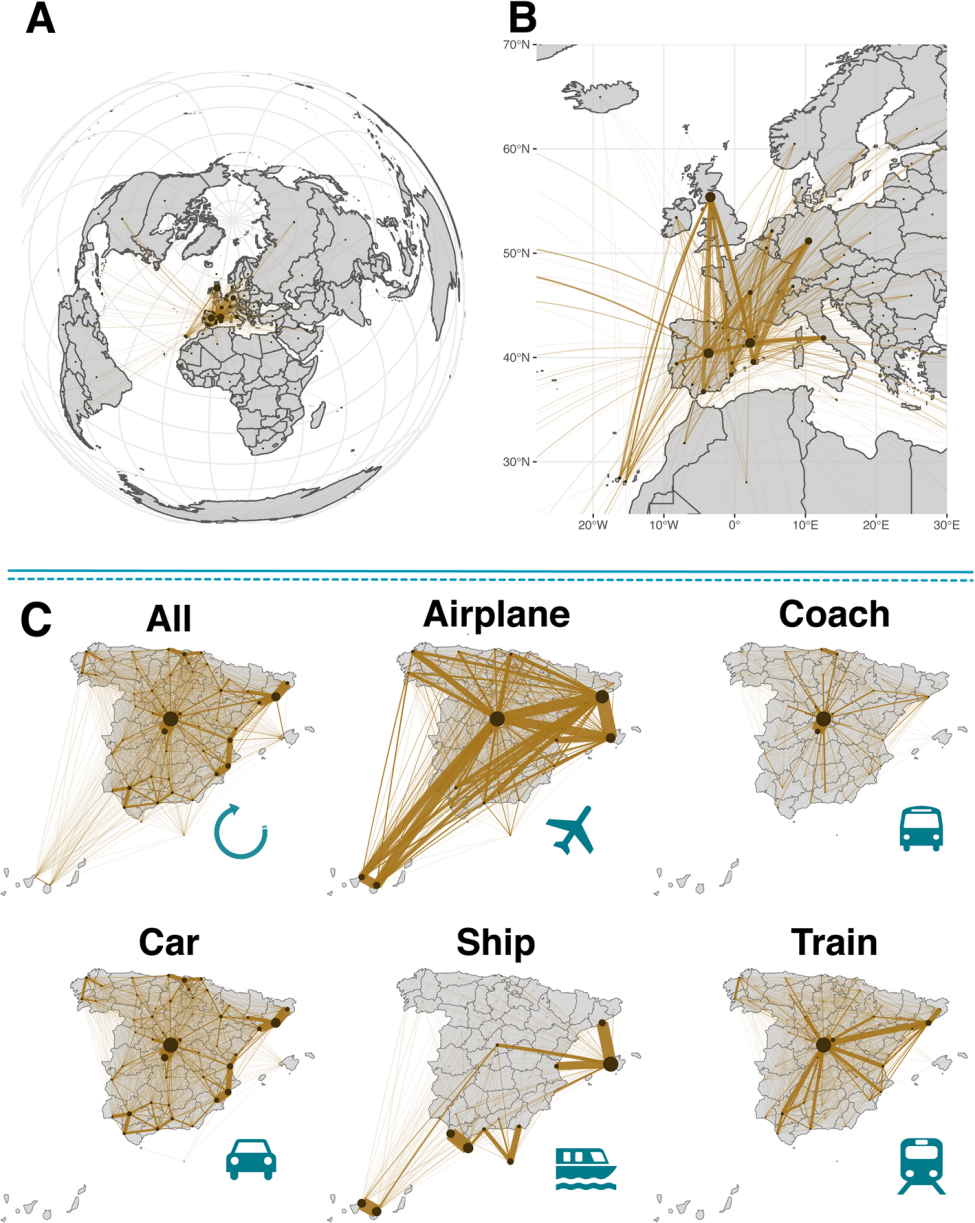
Mobility dynamics in Spain. We use a dataset that includes all possible transportation means, from airplanes to cars. **a**, **b** The international fluxes to Spain. **c** A breakdown of inter-province flows in Spain by transportation mode. The size of the nodes is proportional to the number of individuals leaving the province. Similarly, the width of the links is proportional to the number of individuals using that route. Note that for multimodal travels, the associated mode is the one that corresponds to the largest part of the trip, which explains why there are links from the islands to provinces without ports in the matrix corresponding to maritime trips

To model the mobility of individuals, we use a data-driven approach. Data-driven modeling, at variance with more theoretically inspired methods, has the advantage of allowing the direct implementation and evaluation of realistic containment measures, thus producing scalable and actionable what-if scenarios. To this end, we have obtained the inter-province mobility flows provided by the Ministry of Development of Spain (see Additional file 1: section 1) [16, 17]. Therefore, the minimal spatial unit in our system is a province. Using the information from the mobility matrices that report the origin and destination (OD) of individual movements, at each time step, we sample the number of individuals on the move from each province and distribute them across the country according to the information contained in these OD matrices. Important enough, this dataset not only includes the total number of individuals going from province to province, but it also distinguishes the main transportation means used by the individuals, see Fig. 1. This will allow us to gauge the effect of travel restrictions on different transportation modes.

**Fig. 2:**
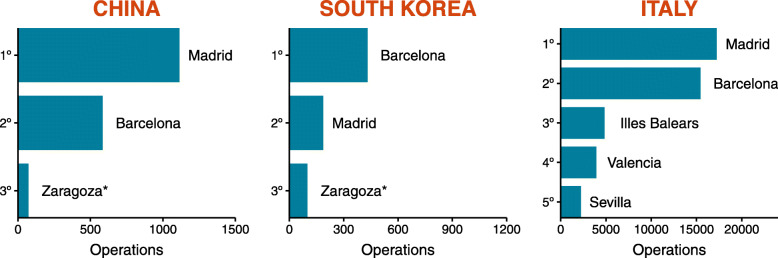
International connections. The number of operations (both passengers and cargo) in 2019 from any airport in China, South Korea, and Italy to each Spanish airport. Only Madrid and Barcelona have direct passenger connections to China and South Korea, whereas Zaragoza only has freight connections to such locations. The destination provinces are ranked according to the likelihood of receiving an infected individual from each country, assuming the order is proportional to the total number of operations

Furthermore, given that the epidemic started abroad, it is important to determine in which province the disease is more likely to be seeded first. As of 1 March, given the global spread and incidence of the epidemic, we take into account that the three countries with more cases are China, South Korea, and Italy and consider that the most plausible route for an infectious individual to reach Spain is by plane. Thus, we collected the number of passengers coming from each country to each Spanish airport in 2019 from the Spanish air navigation manager AENA [15]. Then, we assigned each Spanish airport to its corresponding province and ranked them according to their total number of operations with each country, see Fig. 2. It is worth noticing that the information provided by AENA is already aggregated by country. Thus, this ranking cannot take into account which airports are mostly connected to locations where the outbreak is currently concentrated—e.g., north of Italy. Nevertheless, the ranking constitutes a valid proxy, and a good starting point, to seed the disease.

### Disease dynamics

The dynamics of the disease is governed by an SEIR compartmental model. In this model, individuals are classified according to their health status: susceptible (S) if they are susceptible to catch the disease, exposed (E) if they have been infected but cannot infect other individuals yet, infectious (I) once the latency period is over and the individuals can infect others, and removed (R) when they are either recovered or deceased. Within each province, the transition between compartments results from the following rules, iterated at each time step, corresponding to 1 day:

- Susceptible individuals in province *i* are infected with probability $P_{i}(S\rightarrow I) = 1 - (1-R_{0}/(T_{I}N_{i}))^{I_{i}}$, where *R*_0_ is the reproduction number, *T*_*I*_ is the mean infectious time, *N*_*i*_ stands for the number of individuals in region *i*, and *I*_*i*_ accounts for the number of infectious individuals in such region.
- Exposed individuals become infectious at a rate inversely proportional to the mean latent period, *T*_*E*_.
- Infectious individuals become removed at a rate inversely proportional to the mean infectious period, *T*_*I*_.

In what follows, we parameterize the model according to the latest estimates as of 1 March for the disease parameters [5, 18], namely, *R*_0_=2.5, and a generation time *T*_*g*_=*T*_*E*_+*T*_*I*_=7.5 days resulting from considering *T*_*E*_=5.2 days and *T*_*I*_=2.3 days (in the supplementary material, we report that similar results are obtained for other values of *T*_*g*_, as well as if we allow for pre-symptomatic transmission and higher values of *R*_0_, inline with the most recent estimates as of 13 April, Additional file 1: figures S5-S13). Note that we have not explicitly distinguished between pre-symptomatic, asymptomatic, or symptomatic individuals, being all of them under the category of infectious individuals. Asymptomatic spreading is still under scrutiny. Although there is increasingly more evidence of this transmission route, it is still unknown their relative infectiousness or the amount of actual asymptomatic or mild-symptomatic individuals in the population, with different studies estimating that from 20 to 50% of the infected individuals are asymptomatic [19–24]. It is important to note that it is possible to test positive but develop symptoms several days afterwards, implying that those individuals were pre-symptomatic rather than asymptomatic [22, 25]. Nevertheless, for this early assessment, the inclusion of this type of spreaders would not modify substantially the dynamics under study since we focus on analyzing the most basic strategies to contain or mitigate the outbreak. In any case, once more data is gathered, both about the dynamics related to these subjects and the actual reach of the current outbreak, these new states should be included into these models to properly understand the whole situation.

## Results and discussion

### Quantifying the spatial and temporal evolution of the disease incidence

**Fig. 3:**
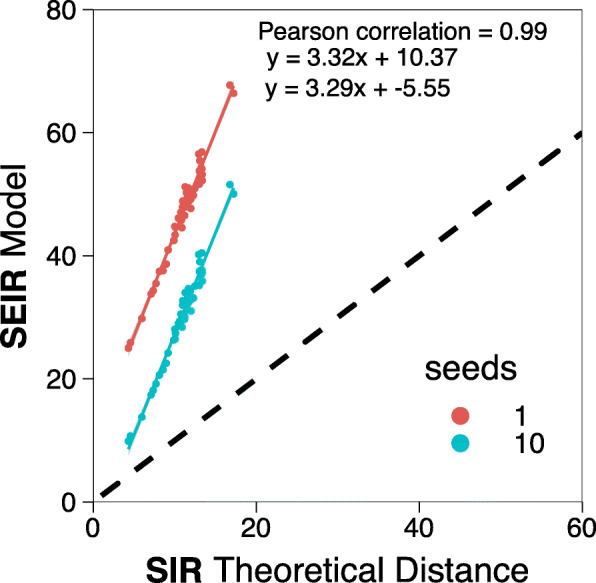
Hitting time in SIR and SEIR models. Comparison between the hitting time obtained after 10^3^ simulations of the SEIR model in our metapopulation scheme, with 1 or 10 seeds initially placed in the province of Barcelona, and the theoretical distance in an SIR metapopulation model

One of the main characteristics of the COVID-19 disease is its long latency times. As of 1 March, the average incubation period has been reported to be 5.2 days [18]. Thus, before proceeding with evaluating the impact of the disease, we first compare the metapopulation model employed here with a classical SIR metapopulation framework. To do so, we use the random-walk effective distance:

1 $$ d_{i(j)}^{\text{RW}}(\delta) = -\ln\left[\left(e^{\delta} I(j|j) - P(j|j)\right)^{-1} p(j)\right]\,,  $$

where *P*(*j*|*j*) is the normalized flow matrix without row and column *j*, *p*(*j*) is the *j* column of *P* with element *j* removed, and *δ* is a dimensionless parameter that depends on the infection, recovery, and mobility rates [26]. This quantity, defined for SIR metapopulation models, gives us the expected time that it would take for the disease to reach each subpopulation of the system, also known as the hitting time. In Fig. 3, we compare the hitting time obtained from stochastic simulations of the SEIR metapopulation model with the theoretical distances derived for the simplified SIR model. We can see that the correlation is nearly perfect, implying that the spreading itself is quite similar in both models. However, we find that the hitting times in the SEIR implementation are at least three times larger than the theoretical ones for the SIR scenario (on its turn, stochastic simulations of the SIR model agree very well with the theoretical expectations for the model, see the Additional file 1: Fig. S4). Thus, the addition of the latent state produces a substantial delay on the spreading of the epidemic. This is in line with the fact that the epidemic is thought to have started in mid-November or early December; however, a noticeable number of cases was only reported by early January.

**Fig. 4:**
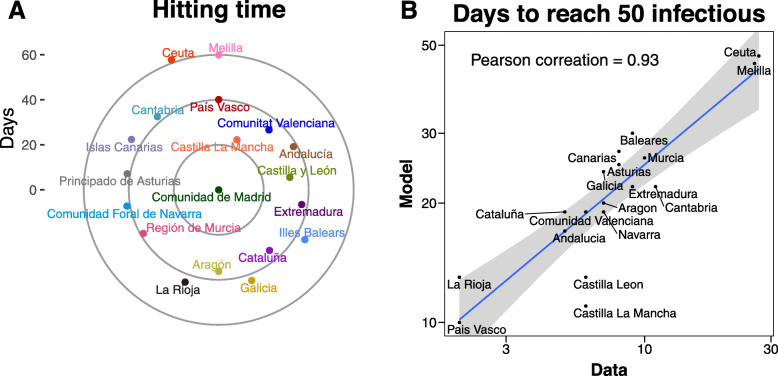
Temporal spreading of the epidemic. In **a**, we show the hitting time obtained when one seed is introduced in Madrid. In **b**, we compare the number of days needed to reach 50 infectious individuals since that number is reached in Madrid. The simulations are seeded with 5 individuals in Madrid, 1 in La Rioja, and 1 in Álava (País Vasco)

Figure 4a shows the expected hitting time for each region (the 52 provinces of Spain are divided in 17 regions, see Additional file 1: section 2) when a disease starts in Madrid with 1 infected individual. As before, the hitting times might seem long, but this is due to the long latent periods of the disease, which is in agreement with the evolution of reported cases in mainland China. We also note that Spanish major cities are expected to be affected by the outbreak in no more than 40 days, although this number is reduced if pre-symptomatic infections are taken into account (see Additional file 1: Fig. S7). In either case, the qualitative behavior is the same since it is a consequence of the underlying data-driven flow dynamics. For instance, the disease would arrive sooner to the Canary Islands, which are situated over 1700 km away from Madrid, than to Ceuta or Melilla, which are only over 500 km away from Madrid, due to the higher amount of traffic between those regions.

To confirm the validity of the model and the data used, in Fig. 4b, we show the expected number of days that it would take for each region to reach 50 infected individuals, compared to the day in which the said number is reached in Madrid. Various clarifications are in order. First, during the month of February, several imported cases from Italy were found, and isolated, all around Spain (see the chronology of the outbreak in Additional file 1: section 3) [27–55] As such, it is too soon to determine the exact number of seeds—and their location. This is out of the scope of this paper. Nevertheless, we have considered a simple approach. We introduced just one single infected individual in Madrid, which has been the hardest-hit region during the outbreak. This leads to a correlation between the time needed to reach 50 infectious individuals in each region and in Madrid of 0.79 (see Additional file 1: Fig. S5). The correlation can be enhanced if we introduce some extra information. For instance, during the first days of March, a large cluster of over 60 cases, shared between the provinces of La Rioja and Álava, was detected and linked to a funeral that took place on 24 February, which was attended by a couple who had recently been to Italy. To take into account this event, we introduce two additional seeds, one in each province, at the beginning of the simulation, which rises the correlation to 0.90 (see Additional file 1: Fig. S5). Furthermore, if we increase the number of initial seeds in Madrid to 5 (to account for the fact that the disease might have arrived there several days before 24 February), the correlation goes up to 0.93, as shown in Fig. 4b. Although different seed choices lead to different results, it is clear that the qualitative behavior of the model holds and is able to reproduce the observed early evolution of the outbreak properly. Furthermore, even though the results in Fig. 4b only match qualitatively the observations, the agreement is much closer if we allow for pre-symptomatic infections (see Additional file 1: Fig. S6), signaling that this type of infections might have been crucial in the early development of the outbreak.

**Fig. 5:**
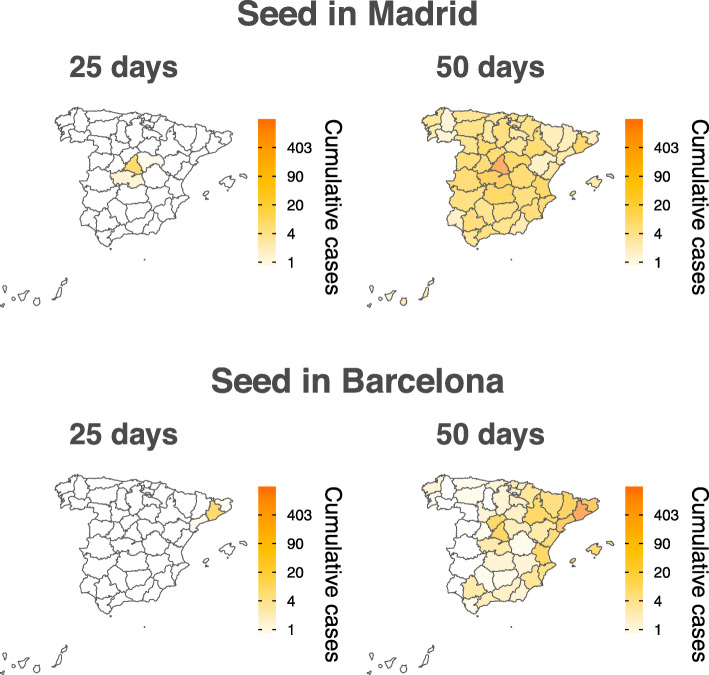
Spatial spreading of the epidemic. Estimated cumulative number of infected individuals within each region when the disease starts with 1 infected individual in Madrid (top row) or in Barcelona (bottom row). The reported values are the median over 10^3^ simulations

Complementarily to Fig. 4, we present in Fig. 5 further results on the temporal and spatial evolution of the disease dynamics. Here, we have computed, through stochastic simulations of the model, the cumulative median number of infected individuals within each region assuming that the disease propagates from Madrid (top row) or Barcelona (bottom row) by initially 1 infectious individual. The results align with the theoretical predictions and highlight the close relationship between the two biggest cities of Spain (Barcelona and Madrid), even though they are relatively far geographically (around 620 km through the shortest path by car). Furthermore, this figure also signals that one of the reasons why the disease might have had such a heavy outcome in Spain is because it started in Madrid. Indeed, under the same conditions, the spatial spread of the disease would have been much lower if it had started in Barcelona by the time the containment measures were put in place by the government. Finally, it is worth remarking two things. First, pre-symptomatic spreading would accelerate the dynamics, although the qualitative geographical distribution of cases should not be affected (see Additional file 1: Fig. S7). Additionally, we also stress the many unknowns that cannot be taken into account yet, such as inflow of infected subjects from abroad. However, as we show next, this data-driven modeling approach allows evaluating the effect of customary containment measures.

### Containment of the epidemic

Our data-driven model is particularly useful to get insights into mobility-mediated transmission dynamics and to evaluate possible countermeasures. Next, we explore diverse containment measures that could be implemented aiming at stopping the large-scale spreading of the disease. First, we analyze the effect of imposing mobility restrictions by limiting traffic flow in the country. We consider six different scenarios that correspond to each transportation mode being shutdown plus another one in which a total reduction of 90% of the overall traffic is imposed. However drastic these measures appear to be, actually, the lockdown declared on 14 March reduced the overall traffic between provinces between 80 and 90% [56]. Nonetheless, as we show below, these measures alone are useless when it comes to completely stop the disease from propagating. Indeed, a significant reduction in the estimated incidence is only obtained when other actions are implemented.

**Fig. 6:**
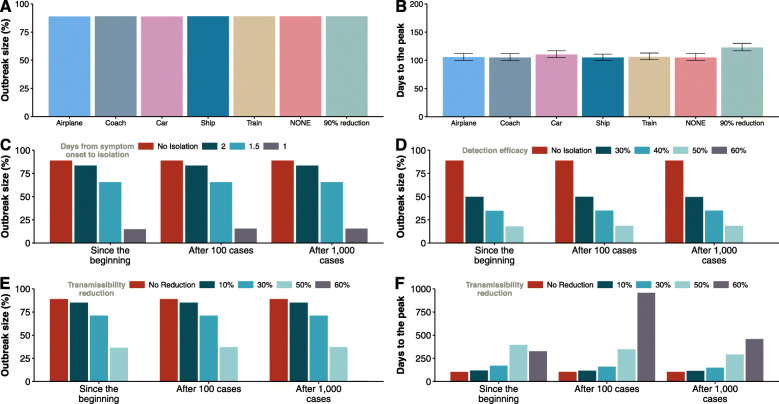
Strategies to mitigate the impact of the disease. **a**, **b** The impact of mobility reduction. **c**–**f** The effect of different measures aimed at reducing the spreading of the epidemic when they are applied since the beginning of the outbreak and after 100 or 1000 cases are detected in the whole country. **a** The fraction of individuals who where affected by the disease by the end of the epidemic. **b** The time from the arrival of the first infected individual to the country until the peak of the epidemic, i.e., the day with the maximum number of infected individuals. In **c**, we evaluate the size of the epidemic if individuals are hospitalized or isolated after a given number of days from the onset of disease symptoms. In **d**, we show the effect of only hospitalizing or isolating a certain fraction of individuals after they experience the first symptoms. In **e**, **f**, we show the size of the epidemic and the time for the disease to peak when transmission is reduced. Note that reducing the transmissibility always delays the spreading, except in situations where the disease dies out, for which the peak occurs earlier. In all cases, the spreading starts with 10 infected individuals in Barcelona

In Fig. 6a, it is observed that the previous measures have no effect on the final size of the epidemic. On the other hand, if we look at the time for the peak of the epidemic to arrive, Fig. 6b, we see some minor effects. In particular, although shutting down most modes of transportation has practically no effect, if all private cars were removed (i.e., they remain confined in their corresponding province), the peak of the epidemic would be delayed by about 7 days. The most effective of the above scenarios of mobility restriction corresponds to a 90% reduction of the overall traffic, when the peak would be delayed over 20 days. This is in agreement with previous studies that have shown that the only sizable effect of travel restrictions is to delay the peak of the epidemic. For instance, it has been claimed that the travel restrictions in Wuhan only delayed the peak of the epidemic by 3 days [5].

Another possibility, instead of limiting the mobility of the overall population, is to be extremely vigilant so as to make it possible to isolate all the cases that start to appear quickly enough. To check the impact of this policy, we have simulated a scenario in which the average number of days that an individual is able to go unnoticed and infect others is reduced from 2.3 to 2, 1.5, and 1 days. In Fig. 6c, we observe that this strategy is much more effective than traffic restrictions. In particular, if we were able to reduce the time since symptoms’ onset to isolation below 1.5 days, the epidemic would be greatly reduced. As a matter of fact, it has been reported [18] that this average number of days went down in China from 4.4 days at the beginning of the outbreak to 2.6 days, which is one of the reasons invoked to explain why the epidemic started to decline in mainland China. In our case, these numbers would be compatible with generation times of 10 or 12.5 days, or with the addition of pre-symptomatic spreading (see Additional file 1: Figs. S9 and S12). As of 13 April, the median time from symptom onset to hospitalization in Spain is 6 days (see Additional file 1: Fig. S2). As such, this measure alone will not currently be able to stop the spreading of the outbreak in Spain.

Furthermore, it is also reasonable to assume that this strategy is not easy to implement in full, either because some individuals could purportedly try to avoid isolation or due to the fact that many infected subjects have mild symptoms similar to a common flu and neither go to the doctor nor report their state. Therefore, we have simulated a slightly different scenario in which individuals are isolated the same day of their symptoms’ onset with a certain efficacy. That is, only a given percentage of the new cases are isolated, while the others are able to roam freely. This framework would also be compatible with having asymptomatic or pre-symptomatic individuals who are able to spread the disease. The equivalence with such hypothetical natural history of the disease in our model is such because we do not apply the prescribed percentage to the total number of infectious individuals, but only to those who have just become infectious; thus, those that escape will remain infectious as if they were asymptomatic until they recover. In Fig. 6d, we show the effect that different percentages of new isolated cases would have on the size of the outbreak. Being able to isolate all individuals, on average, in less than 1 day enables to effectively stop the disease. Yet, the results also show that even if all infectious are not isolated, the size of the outbreak can be greatly reduced.

Lastly, we analyzed the consequences of self-protection measures such as wearing masks, washing more frequently one’s hands, or avoiding crowded places. To mimic these contexts, we simply reduced the effectivity of the transmission by a certain fraction and study the final size of the epidemic, see Fig. 6e. The results show that a large reduction of at least 60% is needed to contain the disease. Interestingly, if we look at the time to the peak of the epidemic, represented in Fig. 6f, we observe that decreasing the transmission not only reduces the size of the outbreak but also delays the peak. Hence, even if this strategy might not be sufficient to completely stop the propagation of the disease in all cases, it could certainly help for preparedness and other clinical responses by delaying the spreading. The exception is when the reduction is very large (in the figure, beyond 60%) as in these cases the peak might occur earlier because the disease dies out.

## Conclusions

It is apparent from all the results obtained for the different scenarios that we have considered that the most cost-effective strategy would be the isolation or quarantine of detected infectious cases, as long as the efficacy of such measure is over 50%. Important for the current debate about the natural history of the disease, this policy would also work if there is a fraction of asymptomatic but infectious individuals in the population. Our results also show that from a practical point of view, a combination of all the analyzed contexts can have second order benefits. As already stressed, containment measures should not only be directed towards a full cut-down of the number of infected cases. Their efficacy is also given by other factors, such as delaying, even if only by a few days, the spreading of the disease. Such delays are always good for preparedness and to have more time for clinical research that can lead to new pharmacological treatments or vaccines. For instance, even if traffic restrictions are not effective on their own, they facilitate the control of the population, and thus, it would be easier to detect infected individuals and treat them. Similarly, self-protection measures and other social-distancing practices delay the spreading of the disease, freeing resources that would allow for a better management of the epidemic, in turn leading to an increase of the efficacy of individual isolation. Closing public places would, in practice, reduce the transmission, which again will lower the total number of infections and thus make them more manageable for the health care system. This also highlights the importance of having a coordinated response system, since simply adopting central measures like imposing mobility restrictions and closing public places is not effective in the middle to long term.

This study also highlights the importance of introducing data-driven mobility patterns of the population. We show that under the same conditions, the spatial spreading of the disease would have been completely different if it had started in Barcelona rather than in Madrid, even if they are both the most important cities in Spain. Furthermore, this result also calls for the need of coordinated containment actions, both at the country and higher levels, to mitigate the spreading of the disease. Indeed, even though a full lockdown of the country would have had a minimal effect, from the spatial point of view, on the situation simulated in Fig. 5 when the disease is seeded in Madrid, the same strategy would have had a great effect if it had started in Barcelona. Yet, if only the province of Barcelona were to be isolated—something that might seem reasonable since in the rest of the provinces the prevalence is fairly low in this hypothetical situation and thus might have been unnoticed by the authorities—that would have not stopped the spreading at all, in line with our observations that mobility reductions on their own delay the peak of the epidemic but do not stop the outbreak.

Our model has several limitations, and some of them could actually be overcome in the near future. Perhaps, the most important one has to do with the inability of current large-scale epidemiological models to fully account for behavioral changes in the population when a disease is evolving. An interesting observation in this regard is that on 9 March, the region of Madrid announced the closure of all schools and universities, including some residence halls. As a consequence, hundreds of students went back to their home regions [38]. This might have accelerated the spreading of the disease since the symptoms in young individuals are quite mild and thus might have transmitted the disease unawerely. Even more, almost 30% of all university students in Madrid come from other Spanish regions [57]. The extent of this effect is for sure something worth exploring in the future and signals how this discipline—in relation with the introduction of behavioral changes—is still in its infancy. Furthermore, as it is the case for the spreading of COVID-19, the information—and more often than desired, misinformation—travels faster than the disease. This produces undesired effects such as a collapse in the emergency rooms at hospitals, a proliferation of information sources that do not provide sensible advices in all cases, anticipated economic loses, and, in general, uncoordinated responses. Therefore, it is a pressing challenge to develop more realistic ways to incorporate in models like the one employed here all these risk-averse responses and reactions. Another limitation of the current study includes the relatively low spatial resolution, which is essentially determined by the data availability. The results, however, indicate that the level of granularity used here is enough to capture mobility patterns and the effects of possible interventions. Finally, we have not considered the temporal and spatial variability of disease parameters, such as the one induced by seasonality (which current estimates signal that will have a small impact on the spreading of the disease due to the huge proportion of the population still susceptible [58]). We have also not included other potentially important characteristics of the host population such as the existence of super spreaders or the age structure, which seems to play a relevant role for this disease, at least in what concerns the case fatality rate. We plan to investigate on all these issues in the near future.

## Supplementary information

**Additional file 1** Data sources and sensitivity analysis on the epidemic parameters. Supporting information of the manuscript Evaluation of the potential incidence of COVID-19 and effectiveness of containment measures in Spain: a data-driven approach.

## Data Availability

The datasets used in this study have been made available at zenodo [59] and can also be downloaded from the original sources [14, 15, 17].

## Abbreviations

AENA: Aeropuertos Españoles y Navegación Aérea (Spanish Airports and Air Navigation)
OD matrix: Origin-destination matrix
SEIR: Susceptible-exposed-infectious-removed compartmental model
SIR: Susceptible-infectious-removed compartmental model
WHO: World Health Organization
