# Supplementary material for: Evaluation of the potential incidence of COVID-19 and effectiveness of containment measures in Spain: a data-driven approach
Source: BMC Med. 2020 May 27;18:157. doi: 10.1186/s12916-020-01619-5 (PMC7250661; doi:10.1186/s12916-020-01619-5)
Supplement: Supplementary file 1 — Additional file 1 Data sources and sensitivity analysis on the epidemic parameters. Supporting information of the manuscript Evaluation of the potential incidence of COVID-19 and effectiveness of containment measures in Spain: a data-driven approach. [file 12916_2020_1619_MOESM1_ESM.pdf]

RESEARCH

Supplementary information for "Evaluation of the potential incidence of COVID-19 and effectiveness of containment measures in Spain: a data-driven approach"

Alberto Aleta[1*] and Yamir Moreno[2,3,1]

*Correspondence: albertoaleta@gmail.com
[1]ISI Foundation, Via Chisola 5, 10126 Torino, Italy
Full list of author information is available at the end of the article

Contents

1 Movement data 1

2 Geographic and Demographic data 2

3 Chronology of the disease in Spain from late January to the declaration of the state of alarm on the 14th of March 3

4 Data collection in Spain 4

5 Effective distances for epidemic spreading 7

6 Robustness of the results 8

1 Movement data

The inter-province flow matrices include the number of individuals that move from province to province in Spain for several days of October 2017, figure S1. The data also includes the main mode of transport used by those individuals, as well as other characteristics such as the period of the day when the travel started. In our case, we have collected the matrices from the last two weeks of October and averaged and symmetrized them.

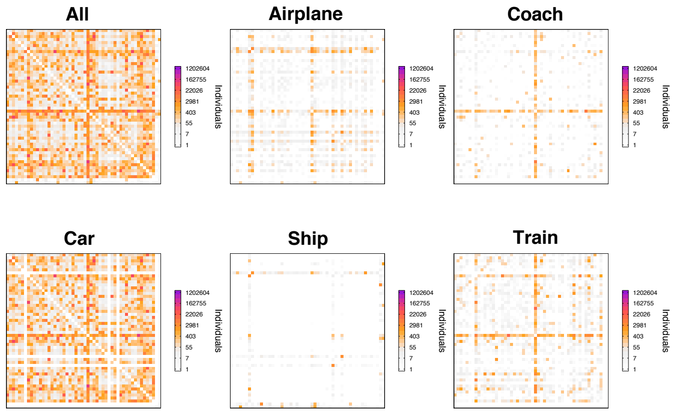

Figure S1 Average flow matrices in the last two weeks of October 2017.

In particular, the matrices were built using mobile phone data coming from 16 million users as part of a pilot project of the Ministry of Development. The objective of the project was to evaluate the potential of such data sources. The outcome was considered successful, since the correlation between the movement predicted by this data and the real data that the Ministry has available (such as the number of passengers traveling by plane or train) is highly correlated. As a consequence, the National Institute of Statistics announced in late 2019 that they will follow this methodology in a larger study in which all the spanish communication companies will participate [1]. For a deeper discussion on the characteristics of these matrices we refer the reader to the original source (in Spanish) [2].

2 Geographic and Demographic data

The upper level of administrative division in Spain is denominated *Comunidad Autónoma*. There are 15 of such "autonomous communities" in mainland Spain, one in the islands of the mediterranean sea (Balearic Islands) and one for the islands in the Atlantic Ocean (Canary Islands). Besides, there are two autonomous cities (*ciudades autónomas*) in the north of Africa, Ceuta and Melilla. The next administrative division is the province (*provincia*). There are 47 provinces in mainland Spain, 1 in the Balearic Islands and 2 in the Canary Islands, plus the two autonomous cities making up the 52 subpopulations considered in our model. The number of inhabitants of each province varies a lot, from over 5 millions in Madrid and Barcelona to less than 100,000 individuals in Ceuta and Melilla. We collected

the number of inhabitants of each province from the data provided by the Spanish Statistical Office [3].

There are 45 airports in Spain (including the heliports in Algeciras and Ceuta), although the majority of them only have connections to other Spanish or European airports. As with the provinces, the traffic in these airports varies widely, from over 50 million passengers in Madrid and Barcelona to less than 2,000 in Albacete and Huesca. The information regarding Spanish airports is provided by the Spanish air navigation manager (AENA) [4].

3 Chronology of the disease in Spain from late January to the declaration of the state of alarm on the 14th of March

In the following we present a brief chronology of the events relevant to our discussion leading to the declaration of the state of alarm on the 14th of March [5, 6].

- 31st of January: first case detected and isolated in the Canary Islands.
- 9th of February: second case detected and isolated in the Balearic Islands.
- 25th of February: first confirmed cases in the peninsula. The total number of confirmed cases in Spain was 5.
- 3th of March: first confirmed death due to COVID-19. The patient died on the 13th of February but the cause was identified after a necropsy of the body [7]. The total number of confirmed cases was 190.
- 6th of March: more than 60 cases in the provinces of La Rioja (La Rioja) and Álava (País Vasco) were confirmed and linked to a funeral that took place in Álava between the 23rd and 24th of February, to which a couple that had recently been in Italy assisted [8, 9].
- 7-8th of March: during the weekend, the authorities do not report any information related to the evolution of the COVID-19 outbreak on their website, although they did it on Twitter and in a press conference [10]. The total number of confirmed cases on the 6th of March was 376.
- 9th of March: the number of confirmed cases rises up to 1001. Almost 70% of them were shared between Madrid, La Rioja and País Vasco. Madrid on its own accounted for 50% of the cases and declares the closure of all schools and universities. The same measure is applied in Álava, which was further

extended to the rest of the region (País Vasco) on the 12th of March. La Rioja announces the same measure one day later [11, 12, 13].

- 10th of March: direct flights between Italy and Spain are suspended [14]. The total number of confirmed cases was 1622.
- 11th of March: the WHO declares the outbreak a global pandemic [15]. The closure of universities and some university residences leads to the return of hundreds of students to their home regions [16]. The total number of confirmed cases was 2128.
- 12th of March: the president announces some economic measures aimed to minimize the effect of the epidemic [17]. All the regions in Spain declare the closure of schools and universities, although it is applied at different dates [18]. The total number of confirmed cases was 2950.
- 13th of March: Madrid and Pais Vasco enforce further closures. The president of the goverment announces that the cabinet council will declare the state of alarm on the 14th [19]. The total number of confirmed cases was 4209.
- 14th of March: the cabinet council declares the state of alarm and sets the country on lockdown [20]. The total number of confirmed cases was 5753.

4 Data collection in Spain

The current burden of the disease in Spain, as well as in several other countries, is unknown. Estimates from China and Iceland show that 90% of the actual cases go unnoticed [21, 22]. As such, fitting models to the current number reported by the authorities is not a valid approach, unless this huge amount of undetected cases is taken into account somehow. Furthermore, in the particular case of Spain the early data collection has been noisy. For instance, on the 6th of March (Friday) the situation in Spain was considered under control, something that changed completely on the 9th of March (Monday). Yet, the Ministry of Health did not update the number of cases in their website during the weekend (even though they gave a press conference and released the data on Twitter) [10]. In the following, we address some of the issues that can be found in the current estimates and that probably will not be solved in the near future. In the future, forensic data analyses will be key to understand the real spreading of the outbreak in Spain.

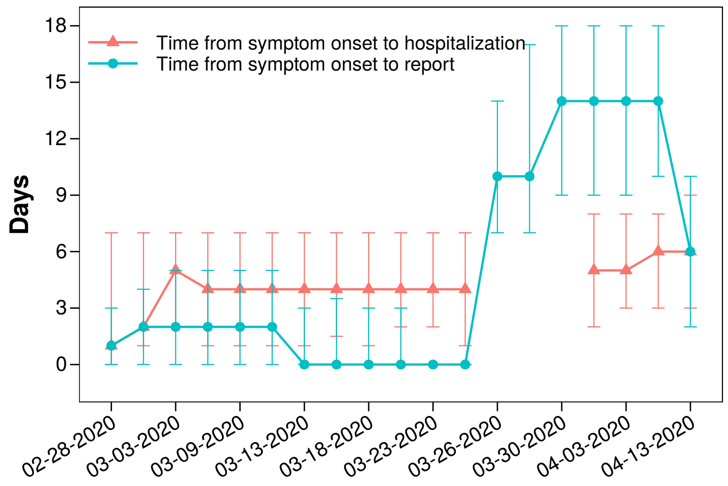

Figure S2 Median and interquartile range of the number of days elapsed between symptom onset and hospitalization (triangles) and reporting to the authorities (circles).

One example of the many unknowns in the current data is the huge delay that exists between symptom onset, detection, reporting to the regions and the final report to the central authorities. In figure S2, we show the estimated number of days elapsed between symptom onset and hospitalization and between symptom onset and reporting the case to the central authorities, as estimated by the Ministry of Health (which can be found in the bulletins they publish every few days [6]). Initially, the time elapsed between the symptoms onset and the report to the authorities was estimated to be around 2 days, implying that the numbers actually seen by the authorities had a delay of at least 2 days, which added to the long period that passes between the infection and the symptom onset (over 5 days) implies that the infections took place almost 7 days before. However, recently, the estimated time from symptom onset to report has been increased to 15 days. This implies that many cases that are currently being added correspond to infections that actually took place several days before. In other words, the numbers provided today by the government are, at least, a consequence of infections occurring more than 20 days ago.

Furthermore, each region collects its data and then reports it to the central government. However, not all regions follow the same methodology. For instance, initially the region of Madrid reported the cumulative number of cases, hospitalization, etc.,

while Castilla y León and País Vasco reported the current number [23]. Even more, the government asks each region to report their numbers by 9pm and releases the data on the next day at 11.30. However, some regions sometimes report their numbers after 9pm - both to the government and publicly - and the Ministry of Health does not include them correctly on the next day. This not only distorts the official data, but also introduces a lot of noise since each region also publishes their data on their own [24]. On the 17th of April, the government has issued a new order through the Official State Gazette to homogenize the way data is collected all over Spain, specially those related to deaths [25].

There are a lot of issues with the current estimates of the number of deaths due to COVID-19. As of 17th of April, Calatuña has modified the way they count the deceased due to coronavirus and, as a consequence, the central government has decided to not include their new numbers [26]. The Daily Mortality Monitoring System (MoMo) has found that the excess mortality since early March is much larger even if the COVID-19 cases are taken into account, signaling that many deaths due to COVID-19 are not being currently accounted for [27]. Some estimates increase the actual number of deaths by 50% [28, 29]. On the 13th of April the Supreme Court of the region of Castilla y León ordered the authorities of the region to include also the deceased that are suspected to have died due to the COVID-19, even if they were not tested [30].

Lastly, the number of total tests performed is not public, although some regions publish their numbers every day, showing that the testing capacities in the country have been steadily increasing during this outbreak. In press conferences, the data released by the Ministry of Health ranges from 15,000 to 20,000 daily tests in the whole country [31], numbers that - it is claimed - have increased now to over 40,000 daily tests on the 17th of April [32]. This, of course, has a major impact on the actual number of infected individuals in the population. We report the number of total cases until the end of March provided by RTVE (the national state-owned public-service radio and television broadcaster in Spain) in figure S3 [5]. Note that the fact that the Ministry did not update the cases on the 7th and 8th of March can still be observed, even though the Ministry has already fixed that on their website [33].

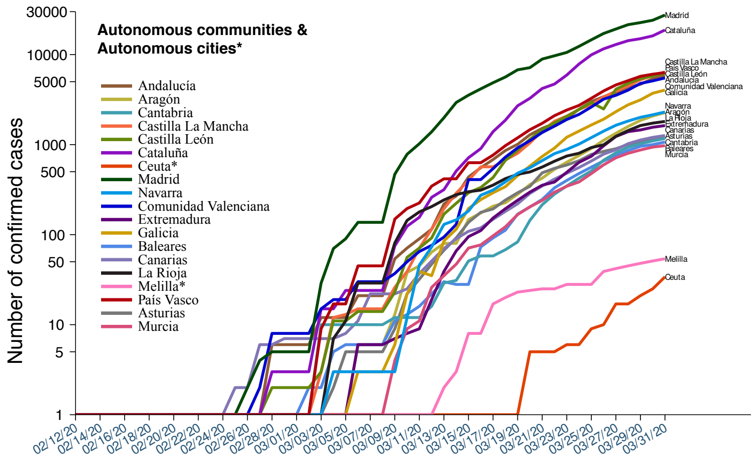

Figure S3 Total number of confirmed cases in each region from early February to late March. Information extracted from [5].

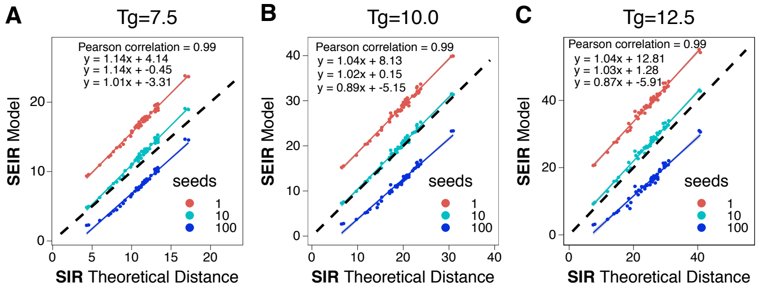

Figure S4 Relationship between the hitting time in a simulation of SIR dynamics in our metapopulation and the theoretical one for different number of seeds and three values of the generation time T_g (in days).

5 Effective distances for epidemic spreading

To ensure that the hitting time is able to predict correctly the spreading in our metapopulation, we first implement an SIR model and study the effect of using a different number of seeds, figure S4. Regardless on the number of seeds, the correlation between the obtained values and the theoretical ones is really excellent. However, the actual value depends on the number of initial seeds. Thus, without any modification this measure can help us determine the arrival order, i.e., to which provinces the disease should arrive first, but not the precise time. Nonetheless, depending on the number of seeds, a small correction could be applied.

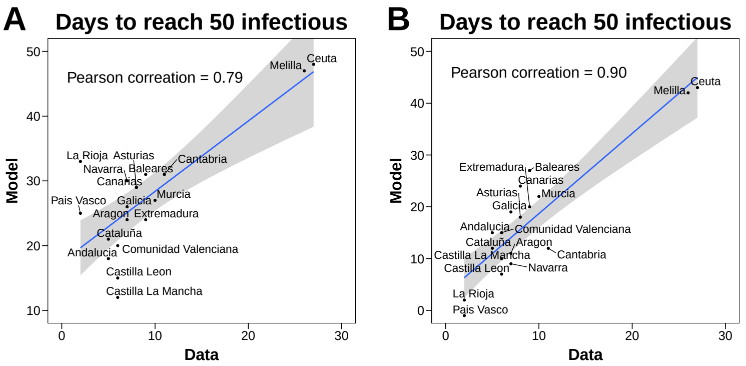

Figure S5 Days needed to reach 50 infectious individuals in each region after said number is reached in Madrid. In panel A, the simulation is seeded with one infectious individual in Madrid. In panel B, the simulation is seeded with three infectious individuals, one in Madrid, one in Álava and one in La Rioja.

6 Robustness of the results

As it is not clear yet what are the final disease parameters that affect the predictions about the spreading of the coronavirus, we here provide quantitative evidence that the results reported in the main text still hold for other values. As a summary, increasing R_0 or adding pre-symptomatic infectivity accelerates the spreading of the disease. On the other hand, larger generation times facilitate the control of the epidemic.

In figure S5 we show the comparison between the progression of the disease in our model and in the data for different seed choices. Clearly, incorporating the information related to the unusual event that took place in Álava and La Rioja improves the power of the model. Still, quantitatively the results of the model suggest a much slower spreading than the real one. In figure S6 we show the effect of adding pre-symptomatic spreading. To do so, we reduce the incubation period from 5.2 days to 3.0 days. Clearly, spreading is highly accelerated and much more compatible with the actual numbers. As such, pre-symptomatic spreading has probably been one of the key elements in this outbreak. We further explore the role of pre-symptomatic infectiousness in figure S7. Once again, we observe that the particularities of the Spanish transportation network accelerate the spreading if the infection starts in Madrid.

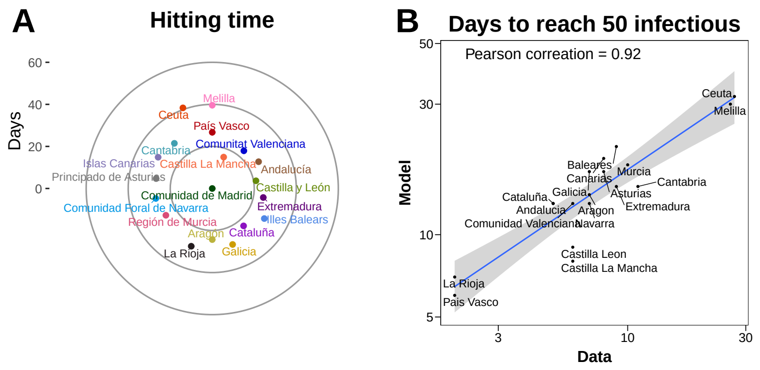

Figure S6 Effects in the early spreading of the introduction of pre-symptomatic infections, $R_0 = 3.0$ and $T_E = 3.0$ days. Panel A: hitting time when the disease starts in Madrid. Panel B: correlation between the speed of the spatial spreading of the disease in the model and the real data (the model is seeded as in the main text, i.e. 1 seed in La Rioja, 1 seed in Ávila and 5 seeds in Madrid).

In figures S8, S9 and S10 show results obtained for $T_g = 7.5$ days, $T_g = 10$ days and $T_g = 12.5$ days, $R_0 = 2.5$ and $T_E = 5.2$ days. As it can be seen, the conclusions of the manuscript hold, and in most of the cases, also quantitatively in terms of relative variation with respect to the scenario in which no measures are implemented. Similarly, in figures S11, S12 and S13 we report the same analysis using higher values of R_0 and lower values of T_E, mimicking the introduction of pre-symptomatic infectiousness.

Author details

[1]ISI Foundation, Via Chisola 5, 10126 Torino, Italy. [2]Institute for Biocomputation and Physics of Complex Systems (BIFI), University of Zaragoza, 50018 Zaragoza, Spain. [3]Department of Theoretical Physics, University of Zaragoza, 50018 Zaragoza, Spain.

References

1. INE Will Track the Mobile Phones of All Spain for 8 Days (El INE Seguirá la Pista de Los Móviles de Toda España Durante Ocho Días). (in Spanish, accessed February 2020). https://elpais.com/economia/2019/10/28/actualidad/1572295148_688318.html
2. Studying interprovince mobility of passengers using Big Data. Technical report, Ministry of Development of Spain (2019). https://observatoriotransporte.mitma.gob.es/estudio-experimental
3. Spanish Statistical Office. (accessed February 2020). https://www.ine.es/
4. Spanish Air Navigation Manager, AENA. (accessed February 2020). http://www.aena.es
5. The Map of Coronavirus in Spain (El Mapa del Coronavirus en España). (in Spanish, accessed April 2020). https://www.rtve.es/noticias/20200417/mapa-del-coronavirus-espana/2004681.shtml
6. Bulletins Released by the Spanish Ministry of Health. (in Spanish, accessed April 2020). https://www.isciii.es/QueHacemos/Servicios/VigilanciaSaludPublicaRENAVE/EnfermedadesTransmisibles/Paginas/InformesCOVID-19.aspxl
7. Valencia Registers the First Death of a Patient with Coronarivus in Spain (Valencia Registra la Primera Muerte de Un Paciente Con Coronavirus en España). (in Spanish, accessed April 2020).

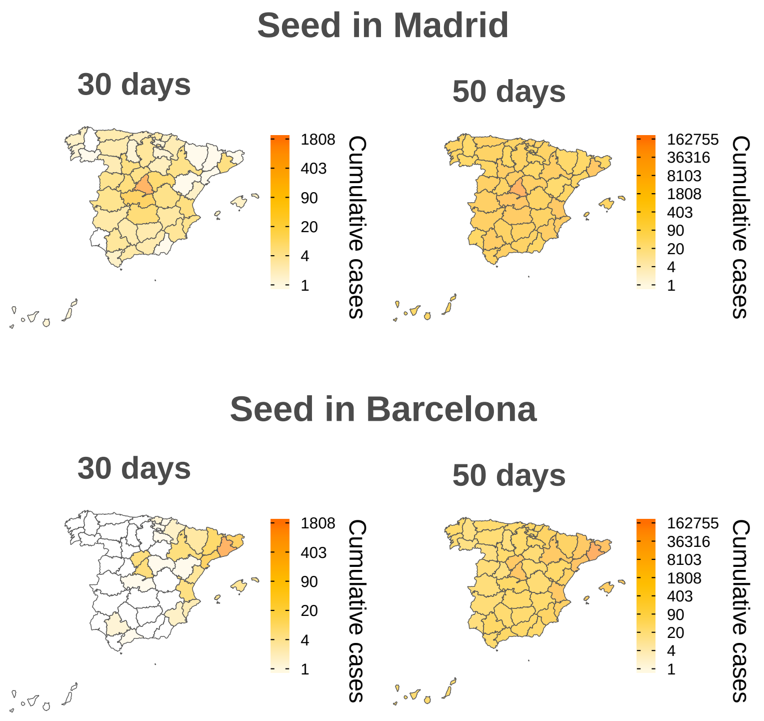

Figure S7 Spatial spreading of the outbreak if it is seeded with 1 infectious individual in Madrid (top row) or Barcelona (bottom row) with pre-symptomatic infections ($R_0 = 3.0$ and $T_E = 3.0$ days)

https://www.abc.es/espana/comunidad-valenciana/abci-hombre-habia-contagiado-coronavirus-murio-13-febrero-valencia-202003032010_noticia.html

8. Chronology of the Coronavirus Epidemic in Spain in Only One Month and a Half (Cronología de la Epidemia de Coronavirus en España en Tan Solo Mes Y Medio). (in Spanish, accessed April 2020). https://elpais.com/sociedad/2020-03-09/cronologia-de-la-epidemia-de-coronavirus-en-espana-en-tan-solo-mes-y-medio.html
9. The Camarón Familiy: the Funeral that Ended in Quarantine (La Familia del 'Camarón': el Funeral Que Acabó en Cuarentena). (in Spanish, accessed April 2020). https://www.larioja.com/la-rioja/funeral-acabo-cuarentena-20200310235850-ntvo.html
10. The Goverment Did Not Stop Counting the Infected Nor the Deceases Due to Coronarivus Between the 6th and 9th of March (El Gobierno No Paró de Contabilizar Los Afectados Ni Fallecidos Por Coronavirus Entre el 6 Y el 9 de Marzo). (in Spanish, accessed April 2020). https://www.newtral.es/bulo-gobierno-cifras-afectados-fallecidos-coronavirus-8-marzo/20200327/
11. La Rioja Freezes Educational Activity, from Kindergarden to University, for 15 Days (La Rioja Paraliza la Actividad Educativa, de las Guarderías a la Universidad, Durante 15 Días). (in Spanish, accessed April 2020). https://www.larioja.com/la-rioja/directo-consejo-gobierno-20200310105327-nt.html
12. All Schools and the University Get Closed Due to Covid-19 (Cierran Todos Los Colegios Y la Universidad de Vitoria Por el Covid-19). (in Spanish, accessed April 2020). https://www.lavanguardia.com/local/paisvasco/20200309/474041388450/labastida-cierra-colegios-coronavirus.html
13. Euskadi Imposes the Closure of All Schools to Stop the Coronavirus (Euskadi Decreta el Cierre de Todos Sus

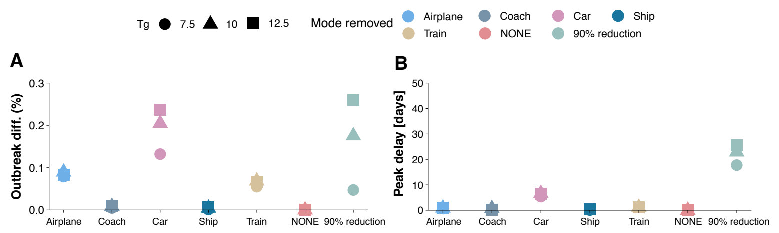

Figure S8 Sensitivity analysis with respect to the value of T_g. The figure shows the results displayed in Figure 6A and 6B but including $T_g = 10$ days and $T_g = 12.5$ days. $R_0 = 2.5$.

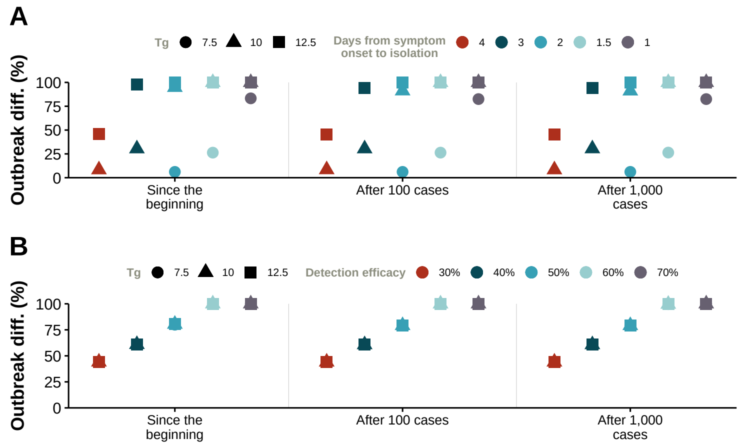

Figure S9 Sensitivity analysis with respect to the value of T_g. The figure shows the results displayed in Figure 6C and 6D but including $T_g = 10$ days and $T_g = 12.5$ days. $R_0 = 2.5$.

Colegios Para Frenar el Coronavirus). (in Spanish, accessed April 2020). https://www.elindependiente.com/politica/2020/03/12/euskadi-decreta-el-cierre-de-todos-sus-colegios-para-frenar-el-coronavirus/

14. Official State Gazette (Boletín Oficial de Estado) of the 10th of March of 2020. (in Spanish, accessed April 2020). https://www.boe.es/boe/dias/2020/03/10/
15. WHO Director-General's Opening Remarks at the Media Briefing on COVID-19 - 11 March 2020. (accessed April 2020). https://www.who.int/dg/speeches/detail/who-director-general-s-opening-remarks-at-the-media-briefing-on-covid-19---11-march-2020
16. Hundreds of University Students Leave Madrid Due to the Coronavirus (Cientos de Universitarios Abandonan Madrid Por el Coronavirus). (in Spanish, accessed April 2020). https://elpais.com/espana/madrid/2020-03-11/exodo-universitario-de-madrid-al-resto-de-provincias.html
17. The President Says that the Goverment Will Not Skimp on Efforts to Reduce at Most the Social and Economic Consequences of the Coronarivus (El Presidente Afirma Que el Gobierno No Escatimará Esfuerzos Para Reducir al Máximo las Consecuencias Sociales Y Económicas del Coronavirus). (in Spanish, accessed April 2020). https://www.lamoncloa.gob.es/consejodeministros/resumenes/Paginas/2020/120320-consejo-extra.aspx
18. 9.5 Million Students Will Be Two Weeks Without Lectures in Spain (9,5 Millones de Estudiantes Se Quedan Dos Semanas Sin Clase en España). (in Spanish, accessed April 2020). https://elpais.com/sociedad/2020-03-12/suspendidas-las-clases-en-todos-los-centros-educativos-de-euskadi.html

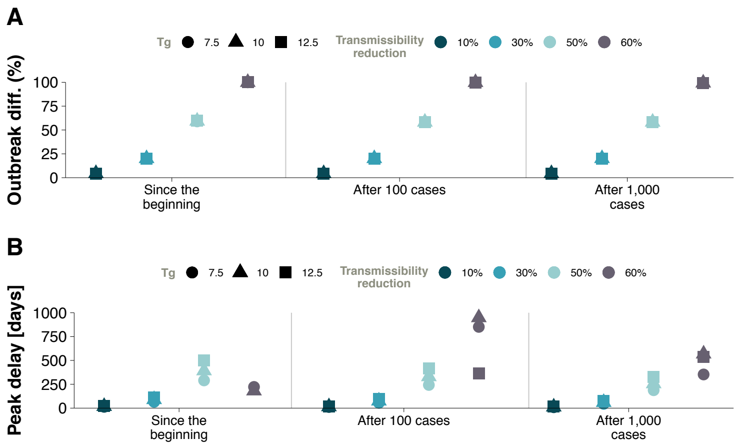

Figure S10 Sensitivity analysis with respect to the value of T_g. The figure shows the results displayed in Figure 6E and 6F but including $T_g = 10$ days and $T_g = 12.5$ days. $R_0 = 2.5$.

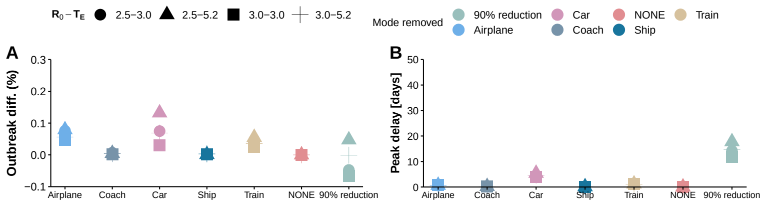

Figure S11 Sensitivity analysis with respect to the value of R_0 and the introduction of pre-symptomatic infectiousness. The figure shows the results displayed in Figure 6A and 6B but including $R_0 = 2.5$ and $T_E = 3.0$ days (circles), $R_0 = 2.5$ and $T_E = 5.2$ days (triangles), $R_0 = 3.0$ and $T_E = 3.0$ days (squares) and $R_0 = 3.0$ and $T_E = 5.2$ days (crosses). In all cases $T_I = 2.3$ days.

19. Pedro Sánchez Announces the Declaration of the State of Alarm (Pedro Sánchez Anuncia la Declaración del Estado de Alarma). (in Spanish, accessed April 2020). https://www.lamoncloa.gob.es/multimedia/videos/presidente/Paginas/2020/130320-sanchez-declaracio.aspx
20. The Goverment Informs that It Is the Unique Authority in Spain, Limits Movements and Closes Shops (El Gobierno Informa de Que Es la única Autoridad en Toda España, Limita Los Desplazamientos Y Cierra Comercios). (in Spanish, accessed April 2020). https://elpais.com/espana/2020-03-14/el-gobierno-prohibe-todos-los-viajes-que-no-sean-de-fuerza-mayor.html
21. Stock, J.H., Aspelund, K.M., Droste, M., Walker, C.D.: Estimates of the Undetected Rate among the SARS-CoV-2 Infected using Testing Data from Iceland. medRxiv (2020). doi:10.1101/2020.04.06.20055582
22. Li, R., Pei, S., Chen, B., Song, Y., Zhang, T., Yang, W., Shaman, J.: Substantial undocumented infection facilitates the rapid dissemination of novel coronavirus (SARS-CoV2). Science, 3221 (2020). doi:10.1126/science.abb3221
23. More Mess with the Official Figures: the Data from Each CCAA Are Not Homogeneous (Más Lío Con las Cifras Oficiales: Los Datos de las CCAA No Son Homogéneos Entre Sí). (in Spanish, accessed April 2020). https://www.elconfidencial.com/espana/2020-03-25/coronavirus-cifras-oficiales-comunidades-autonomas_2515836/
24. Why the Number of Deceased in Catalunya Reported by Moncloa and the Govern do Not Mach? (¿Por Qué No

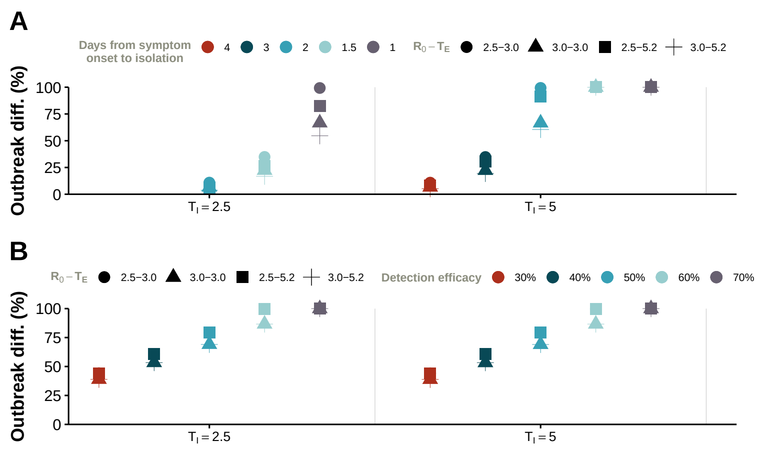

Figure S12 Sensitivity analysis with respect to the value of R_0 and the introduction of pre-symptomatic infectiousness. The figure shows the results displayed in Figure 6C and 6D but including $R_0 = 2.5$ and $T_E = 3.0$ days (circles), $R_0 = 3.0$ and $T_E = 3.0$ days (triangles), $R_0 = 2.5$ and $T_E = 5.2$ days (squares) and $R_0 = 3.0$ and $T_E = 5.2$ days (crosses).

Coincide el Dato de Muertes en Catalunya Que Dan Moncloa Y Govern?). (in Spanish, accessed April 2020). https://www.elnacional.cat/es/politica/coronavirus-muertos-espana-catalunya_484239_102.html

25. Madrid Insists on Changing the Way of Counting Covid-19 Cases (Madrid Insiste en Cambiar la Forma de Recuento del Covid-19). (in Spanish, accessed April 2020). http://www.telemadrid.es/programas/telenoticias-1/Madrid-insisten-cambiar-recuento-Covid-19-2-2223397651--20200417025516.html

26. The Ministry of Health Does Not Include in the Total Numbers the 3,242 New Deceased Counted in Catalunya Even Though They Asked for the Data (Sanidad No Incluye en el Recuento Global 3.242 Nuevos Muertos Contabilizados en Catalunya Pese a Que Pidió Esos Datos). (in Spanish, accessed April 2020). https://www.lavanguardia.com/vida/20200416/48559726872/casos-recuento-fallecidos-sanidad-salut.html

27. Mortality Monitoring System (MoMo) Bulletins. (in Spanish, accessed April 2020). https://www.isciii.es/QueHacemos/Servicios/VigilanciaSaludPublicaRENAVE/EnfermedadesTransmisibles/MoMo/Paginas/Informes-MoMo-2020.aspx

28. The 10,176 Deads Not Counted that Are Yet to Surface: the Data of Spain by Region (Los 10.176 Muertos No Contabilizados Que Quedan Por Aflorar: Los Datos de España Por Comunidades). (in Spanish, accessed April 2020). https://www.elespanol.com/reportajes/20200417/muertos-no-contabilizados-quedan-aflorar-espana-comunidades/482953155_0.html

29. The Burials Due to Coronavirus in Castilla - La Mancha Almost Triple the Official Numbers (Los Enterramientos Por Coronavirus en Castilla-La Mancha Casi Triplican Los Datos Oficiales). (in Spanish, accessed April 2020). https://www.elmundo.es/espana/2020/04/06/5e8b4e4021efa01e6e8b4591.html

30. The Supreme Court of Castilla Y León Commands the Registry to Count Also the Suspected Deaths Due to Covid-19 (El TSJ de Castilla Y León Ordena a Los Registros Contabilizar También las Muertes Sospechosas Por Covid-19). (in Spanish, accessed April 2020). https://www.elmundo.es/espana/2020/04/13/5e94925521efa06d148b460e.html

31. The Health Ministry Hides the Number of Tests Performed in Spain (Sanidad Oculta el Número Total de Test Realizados en España). (in Spanish, accessed April 2020). https://www.elmundo.es/ciencia-y-salud/salud/2020/04/16/5e976353fc6c8397308b45eb.html

32. Coronavirus: Spain Doubles Its Capacity and Reaches 40,000 Tests per Day (Coronavirus: España Duplica Su Capacidad Y Llega a 40.000 Test al Día). (in Spanish, accessed April 2020). https://www.redaccionmedica.com/secciones/sanidad-hoy/coronavirus-espana-duplica-su-capacidad-y-llega-a-40-000-test-al-dia-2240

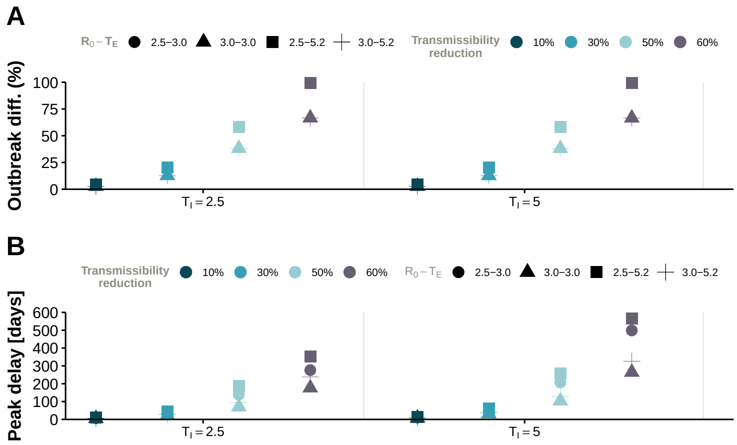

Figure S13 Sensitivity analysis with respect to the value of R_0 and the introduction of pre-symptomatic infectiousness. The figure shows the results displayed in Figure 6E and 6F but including $R_0 = 2.5$ and $T_E = 3.0$ days (circles), $R_0 = 3.0$ and $T_E = 3.0$ days (triangles), $R_0 = 2.5$ and $T_E = 5.2$ days (squares) and $R_0 = 3.0$ and $T_E = 5.2$ days (crosses).

33. Official Data Portal of the Spanish Ministry of Health. (in Spanish, accessed April 2020). https://covid19.isciii.es/
